# Identification of Whey Protein‐Derived Anti‐Obesity Peptides Through 3T3‐L1 Adipocyte Differentiation Assay

**DOI:** 10.1002/fsn3.4529

**Published:** 2024-10-21

**Authors:** Yuma Hirose, Masaki Kurimoto, Naoki Yuda, Miyuki Tanaka

**Affiliations:** ^1^ Innovative Research Institute Morinaga Milk Industry Co., Ltd. Zama Kanagawa Japan

**Keywords:** adipocyte, obesity, peroxisome proliferator‐activated receptor γ, whey peptides

## Abstract

Whey proteins are a rich source of bioactive peptides. Whey protein hydrolysate (WPH) can effectively improve metabolic syndrome and reduce the risk of obesity. Bioactive peptides isolated from various food sources exhibit anti‐obesity effects. However, few reports are available on the identification of anti‐obesity peptides from whey protein. In this study, we aimed to identify anti‐obesity peptides from whey protein. Our findings revealed that WPH suppressed the accumulation of lipid droplets in 3T3‐L1 adipocytes. Anti‐obesity peptides in WPH were identified using amino acid sequencing and LC–MS analysis. Then, the inhibitory effects of the synthetic peptides on adipogenesis were assessed through Oil Red O staining. Two peptides were identified as anti‐adipogenic: LDQW and LKPTPEGDLEIL. Subsequently, real‐time PCR analysis found that several adipogenesis‐related genes, such as peroxisome proliferator‐activated receptor γ, were downregulated in the treatment with these peptides. Furthermore, LDQW and LKPTPEGDLEIL decreased the mRNA expression levels of stearoyl‐coenzyme A desaturase 1 and increased carnitine palmitoyl transferase 1α expression in 3T3‐L1 adipocytes. These findings indicate that LDQW and LKPTPEGDLEIL have anti‐obesity properties and may be beneficial for treating metabolic diseases. This study provides a reference basis for developing new techniques to prevent obesity and related diseases.

## Graphical abstract

In this study, WPH and its derived peptides, LDQW and LKPTPEGDLEIL, reduced lipid accumulation in 3T3‐L1 cells. The inhibitory effect of WPH, LDQW, and LKPTPEGDLEIL on lipid accumulation was mediated through the suppression of adipogenesis‐related mRNA, including PPARγ.

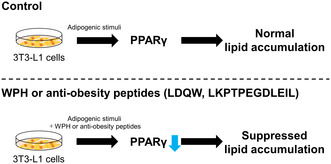

## 1 Introduction

Obesity, a metabolic disorder resulting from a disparity between energy consumption and expenditure, increases the susceptibility of an individual to various illnesses, including diabetes, atherosclerosis, heart disease, and high levels of uric acid. (Blüher 2019; Wang et al. 2022). Obesity may also cause psychological problems such as depression and anxiety (Chu et al. 2019). This condition poses a significant public health and economic threat; the World Health Organization has set a target to halt its increase by 2025 (Ataey et al. 2020). Adipocytes are critical regulators of lipid metabolism and energy balance, and their number and size increase with obesity (Jakab et al. 2021). Therefore, the regulation of adipocyte differentiation may be a critical strategy for preventing obesity.

Preadipocytes mature to form mature adipocytes accompanied by unique morphological and biochemical changes (Cristancho and Lazar 2011). 3T3‐L1 cells and adipose tissue‐derived precursor cells are widely used in research because they can lead to adipocyte differentiation (Tung et al. 2017). The expression levels of proteins involved in lipid transport, accumulation, and metabolism change dynamically during adipocyte differentiation. Furthermore, lipids promote the accumulation of triglycerides and the upregulation of transcription factors, such as peroxisome proliferator‐activated receptor γ (PPARγ) and CCAAT/enhancer‐binding proteins α (C/EBPα) (Cristancho and Lazar 2011; Darlington, Ross, and MacDougald 1998; Tang, Otto, and Lane 2003). PPARγ and C/EBPα then stimulate adipocyte‐specific mRNA activation. Adenosine monophosphate‐activated protein kinase (AMPK) is a crucial regulator of short‐ and long‐term fatty acid oxidation, and it inhibits 3T3‐L1 cell differentiation through the downregulation of adipogenic transcription factors, including PPARγ and C/EBPα (Gao et al. 2008; Lee et al. 2011).

Globally, current research trends are focusing on various food ingredients to prevent and treat obesity (Konstantinidi and Koutelidakis 2019). Several studies have investigated the potential of bioactive peptides derived from food sources such as eggs, soybeans, nuts, fish, meat, and milk (Acquah et al. 2022; Moura et al. 2017; Udenigwe and Rouvinen‐Watt 2015), which could be referred to as functional food ingredients. Bioactive peptides can potentially affect the health and body's function by altering physiological processes and enzymes. For example, these peptides are involved in angiotensin I‐converting enzyme (ACE) inhibition (Duffuler et al. 2022), antioxidant effects (Bao and Wu 2021), and dipeptidyl peptidase‐IV inhibition (Akbarian et al. 2022; Amigo and Hernández‐Ledesma 2020). Research on their various effects is being conducted worldwide. Bioactive peptides isolated and identified from various food sources have anti‐obesity effects. For example, a pentapeptide, RLLPH, derived from hazelnut and a tripeptide, IQN, derived from black soybean, inhibited adipogenesis in 3T3‐L1 cells (Kim et al. 2007; Wang et al. 2020). An octapeptide, DIVDKIEI, derived from tuna fish inhibited C/EBPα and PPARγ expression and reduced triglyceride accumulation in 3T3‐L1 cells (Kim et al. 2015).

Whey protein, which makes up approximately 20% of cow's milk protein (Costa et al. 2021), has garnered interest as a source of bioactive peptides, being the second most prevalent protein following casein (Zhao, Chen, and Ashaolu 2022). Our previous study revealed that skeletal muscle atrophy induced by a protein‐free diet was prevented by whey protein hydrolysate (WPH). Furthermore, WPH accelerated the absorption of amino acids during the postprandial period (Kobayashi et al. 2016). However, the anti‐atrophy effect of WPH cannot be ascribed to only the absorption dynamics of amino acids, and other mechanisms might be involved. Therefore, WPH may contain bioactive peptides that have a suppressive effect on skeletal muscle atrophy.

Numerous pharmacological effects of bioactive peptides derived from whey protein have been documented (Dullius, Goettert, and de Souza 2018), including antioxidants (Sadat et al. 2011), opioid activity (Tyagi et al. 2020), ACE inhibition (Tavares et al. 2011), and antibacterial properties (Pellegrini et al. 2001). In addition, WPH exhibits several metabolic effects, including reducing adipose tissue in aged mice (Ichinoseki‐Sekine et al. 2015). Alpha‐lactalbumin, a whey protein component, hydrolysate improved glucose homeostasis in high‐fat diet (HFD)‐fed mice and suppressed HFD‐induced weight gain (Gao et al. 2018). These studies suggest that WPH reduces obesity and alters lipid metabolism. However, to the best of our knowledge, few reports have focused on the identification of anti‐obesity peptides from whey protein. Therefore, in this study, we evaluated the effect of WPH on lipid metabolism through a 3T3‐L1 adipocyte differentiation assay and identified the bioactive peptide(s) responsible for suppressing adipogenic differentiation.

## 2 Materials and Methods

### 2.1 Chemicals and Materials

WPH (product name WPH‐M), comprising 79.3% protein, 11.7% carbohydrate, 0.4% fat, 4.9% ash, and 3.7% moisture, was manufactured by Morinaga Milk Industries (Tokyo, Japan). WPH was produced enzymatically from whey protein concentrate as previously reported, with an average molecular weight of 418 Da and degree of hydrolysis of 22.9% (Kobayashi et al. 2016).

Dulbecco's modified Eagle's medium (DMEM), penicillin–streptomycin solution, fetal bovine serum (FBS), trifluoroacetic acid (TFA), and formic acid (FA) were purchased from FUJIFILM Wako Pure Chemical Corp. (Tokyo, Japan). Three‐isobutyl‐1‐methylxanthine, dexamethasone, and insulin were purchased from Sigma‐Aldrich Corp. (Saint Louis, MO, USA). High‐performance liquid chromatography (HPLC) grade acetonitrile was purchased from Kanto Chemical Co. Ltd. (Tokyo, Japan). Cadenza CD C18 HPLC columns (10 mm × 250 mm) were purchased from Imtakt Co. Ltd. (Kyoto, Japan), ACQUITY UPLC BEH C18 columns (2.1 mm × 150 mm) and XBridge Peptides BEH C18 columns (2.1 mm × 250 mm) were purchased from Waters Inc. (MA, USA).

Peptides with sequences of ALPM, LDQW, and LKPTPEGDLEIL were obtained via solid‐phase synthesis from BEX Co. Ltd. (Tokyo, Japan), and their purity exceeded 95%.

### 2.2 Purification of WPH

The fractions were purified using HPLC (UltiMate 3000 HPLC; Thermo Fisher Scientific, Waltham, MA, USA). The WPH was dissolved in ultrapure water to 40 mg/mL, filtered using a 0.2‐μm membrane (DISMIC‐25CS, ADVANTEC TOYO KAISHA, LTD., Tokyo, Japan), and then applied to a Cadenza CD‐C18 column. The sample (2 mL) was eluted with 0.1% TFA in water (solvent A) and 0.1% TFA in acetonitrile (solvent B) using the following gradient for 60 min at a flow rate of 3 mL/min: with 2% B for 2 min, 2% to 30% B for 38 min, 30% to 80% B for 10 min, 80% to 2% B for 0.1 min, and held at 2% B for 9.9 min. The elution was monitored at 220 nm. Eight fractions were collected, concentrated, and dried using a vacuum‐rotary evaporator (MiVac Quattro Concentrator; Biopharma Process Systems, Winchester, UK) at 40°C for further analysis in cells.

The fraction with lipid accumulation‐suppressing activity was further purified via HPLC. The dried fractions were dissolved in a 0.1% TFA in water. After centrifuging the mixtures at 239 *g* for 1 min, the soluble supernatant was filtered through a 0.2‐μm filter and added to a Cadenza CD‐C18 column. The sample (2 mL) was eluted using 0.1% TFA in water (solvent A) and 0.1% TFA in acetonitrile (solvent B) using the following gradient for 60 min at a flow rate of 3 mL/min: with 22.5% to 28.5% B for 40 min, 28.5% to 80% B for 10 min, 80% to 22.5% B for 0.1 min, and held at 22.5% B for 9.9 min, and was monitored at 215 nm. The fractions were collected based on major peaks in the chromatogram, concentrated, and dried using a vacuum‐rotary evaporator at 40°C for further analysis in cells. The active fractions were then subjected to further analysis through amino acid sequencing to identify the peptide sequences.

### 2.3 Identification of the Peptide Sequences

The active fractions from reversed‐phase (RP)‐HPLC purification were analyzed using an automated protein sequencer (PPSQ‐50A; Shimadzu, Kyoto, Japan). The phenylthiohydantoin amino acid derivatives were identified at 269 nm after separation on a Wakopak Wakosil PTH‐GR (S‐PSQ) column (250 mm × 2.0 mm; FUJIFILM Wako Pure Chemical Corporation, Tokyo, Japan). Data acquisition were performed using a Shimadzu Techno‐Research, Inc. (Kyoto, Japan).

The peptide sequences analyzed via amino acid sequencing were verified through liquid chromatography‐mass spectrometry (LC–MS) (Q Exactive Focus; Thermo Fisher Scientific, Waltham, MA, USA) paired with an ACQUITY UPLC BEH C18 Column by comparison with synthetic peptides. The fractionated peptides were separated using 0.1% FA in water (solvent A) and 0.1% FA in acetonitrile (solvent B) with the following gradients for 45 min: with 2% to 30% B for 30 min, 30% to 90% B for 5 min, 90% to 2% B for 1 min, and held at 2% B for 9 min. During separation, the column temperature and flow rate were maintained at 40°C and 0.2 mL/min, respectively. MS detection was performed in positive‐ion mode. The optimal ionization conditions were as follows: capillary temperature, 275°C; aux gas flow rate, 10 Arb; sheath gas flow rate, 45 Arb; spray voltage, 3500 V; mass range, from 120 to 1800 m/z; and full MS resolution, 70,000. The characteristics of the expected peptide were compared with those of its synthetic counterpart.

### 2.4 Quantitative Analyses of Peptides in WPH

Quantitative analyses of bioactive peptides in WPH were performed via LC–MS paired with an XBridge Peptides BEH C18 column. WPH was dissolved in ultrapure water and filtered through a 0.2‐μm membrane (1 mg/mL). The sample was separated using 0.1% FA in water (solvent A) and 0.1% FA in acetonitrile (solvent B) with the following gradients for 30 min: with 2% to 40% B for 17.5 min, 40% to 80% B for 3.5 min, held at 80% B for 2 min, 80% to 2% B for 0.5 min, and held at 2% B for 6.5 min. During separation, the column temperature and flow rate were maintained at 40°C and 0.2 mL/min, respectively. MS detection was carried out in positive‐ion mode. The optimal ionization conditions were as follows: capillary temperature, 275°C; aux gas flow rate, 10 Arb; sheath gas flow rate, 45 Arb; spray voltage, 3500 V; MS/MS resolution, 35,000; and collision energy in normalized collision energy mode, 30. ALPM, LDQW, and LKPTPEGDLEIL were quantitated based on the most abundant transition of m/z 431.2 → 247.1, 561.3 → 315.1, and 662.9 → 129.1, respectively. We used synthetic peptides as standards.

### 2.5 Cell Culture

The 3T3‐L1 preadipocytes (JCRB9014) were obtained from the Japanese Collection of Research Bioresources Cell Bank in Osaka, Japan. The cells were cultured at 37°C in a 5% carbon dioxide atmosphere using growth medium (GM) (DMEM supplemented with 100 units/mL penicillin–streptomycin, 10% FBS).

Mature 3T3‐L1 adipocytes were developed in the following manner: after cells reached confluence (designated as Day 0), the medium was changed to a differentiation media (GM supplemented with 1.0 μM dexamethasone, 0.5 mM 3‐isobutyl‐1‐methylxanthine, and 1.0 μg/mL insulin), and the cells were cultured for 2 days. On Day 2 of differentiation, the medium was changed to a GM containing 1.0 μg/mL insulin and WPH, hydrolytic fraction, or peptides, whereafter, the cells were cultured for another 2 days. The medium was changed with a different GM containing 1.0 μg/mL insulin on Day 4, and cells were cultured for another 4–6 days, with the culture medium being changed every 2 days. These 3T3‐L1 adipocytes were later used in other experiments.

### 2.6 Cell Viability Assay

Cell viability was assessed using Cell Counting Kit‐8 (CCK‐8; DOJINDO Laboratories, Kumamoto, Japan) for the WST‐8 assay. The 3T3‐L1 cells in 96‐well plates were treated for 48 h at 37°C with 0.5, 1, and 2 mg/mL of either WPH or hydrolytic fractions at an equivalent concentration of 1 mg/mL of WPH. Following a 2 h incubation of cells with 10 μL of CCK‐8 reagent per well, the absorbance of the samples was measured at 450 nm with the use of a microplate reader. (Corona Electric Co. Ltd., Ibaraki, Japan).

### 2.7 Oil Red O Staining

Intracellular lipid accumulation was measured using a Lipid Assay Kit (Cosmo Bio Co. Ltd., Tokyo, Japan) following the manufacturer's instructions. The stained lipid droplets in cells were dissolved in the dye extraction reagent included in the kit, and the absorbance of the samples was measured at 540 nm using a microplate reader (Corona Electric Co., Ltd., Ibaraki, Japan).

### 2.8 RNA Preparation and Real‐Time PCR

Total RNA was prepared using the NucleoSpin RNA kit (Macherey–Nagel, Düren, Germany). cDNA was synthesized from 2 μg of total RNA using the High‐Capacity cDNA Reverse Transcription Kit (Life Technologies Japan, Tokyo, Japan). Gene expression levels were measured on a QuantStudio 5 Real‐Time PCR System (Thermo Fisher Scientific, Waltham, MA, USA) using Fast SYBR Green Master Mix (Thermo Fisher Scientific, Waltham, MA, USA). The primers (Takara Bio Inc., Shiga, Japan) used for real‐time PCR are listed in Table 1.

**TABLE 1:** Primer sequences used for real‐time PCR.

Gene	Forward primer (5′–3′)	Reverse primer (5′–3′)
GAPDH	TGTGTCCGTCGTGGATCTGA	TTGCTGTTGAAGTCGCAGGAG
PPARγ	GCTGACCCAATGGTTGCTGA	TTCATGAGGCCTGTTGTAGAGCTG
C/EBPα	AGTCGGTGGACAAGAACAGCAAC	CGGTCATTGTCACTGGTCAACTC
FABP4	CCTGGAAGCTTGTCTCCAGTGA	GAATTCCACGCCCAGTTTGA
SCD‐1	TCTTGTCCCTATAGCCCAATCCAG	AGCTCAGAGCGCGTGTTCAA
PRKAA1	TGTGGGCTCTGACATGATGAA	AATCAGGTTACTCTGGGCAAACATA
CPT‐1	CATTGGCCACCAGTTCCATTA	CCAATGGCTGCCACACTCTC
PGC1‐α	TTCCAACCAGTGTGCTGCTC	TGGTCGCTACACCACTTCAATC

### 2.9 Statistical Analysis

To identify the statistical differences between the control and treatment groups, the data were analyzed through unpaired one‐way analysis of variance, followed by Dunnett's test. The data are expressed as the mean ± standard error of the mean (SEM). All statistical analyses were performed using SPSS Statistics 28.0.0.0 (IBM, Armonk, NY, USA). *p*‐values of < 0.05 were considered statistically significant.

## 3 Results

### 3.1 Effect of WPH on Cell Viability and Lipid Accumulation in 3T3‐L1 Adipocytes

**FIGURE 1:**
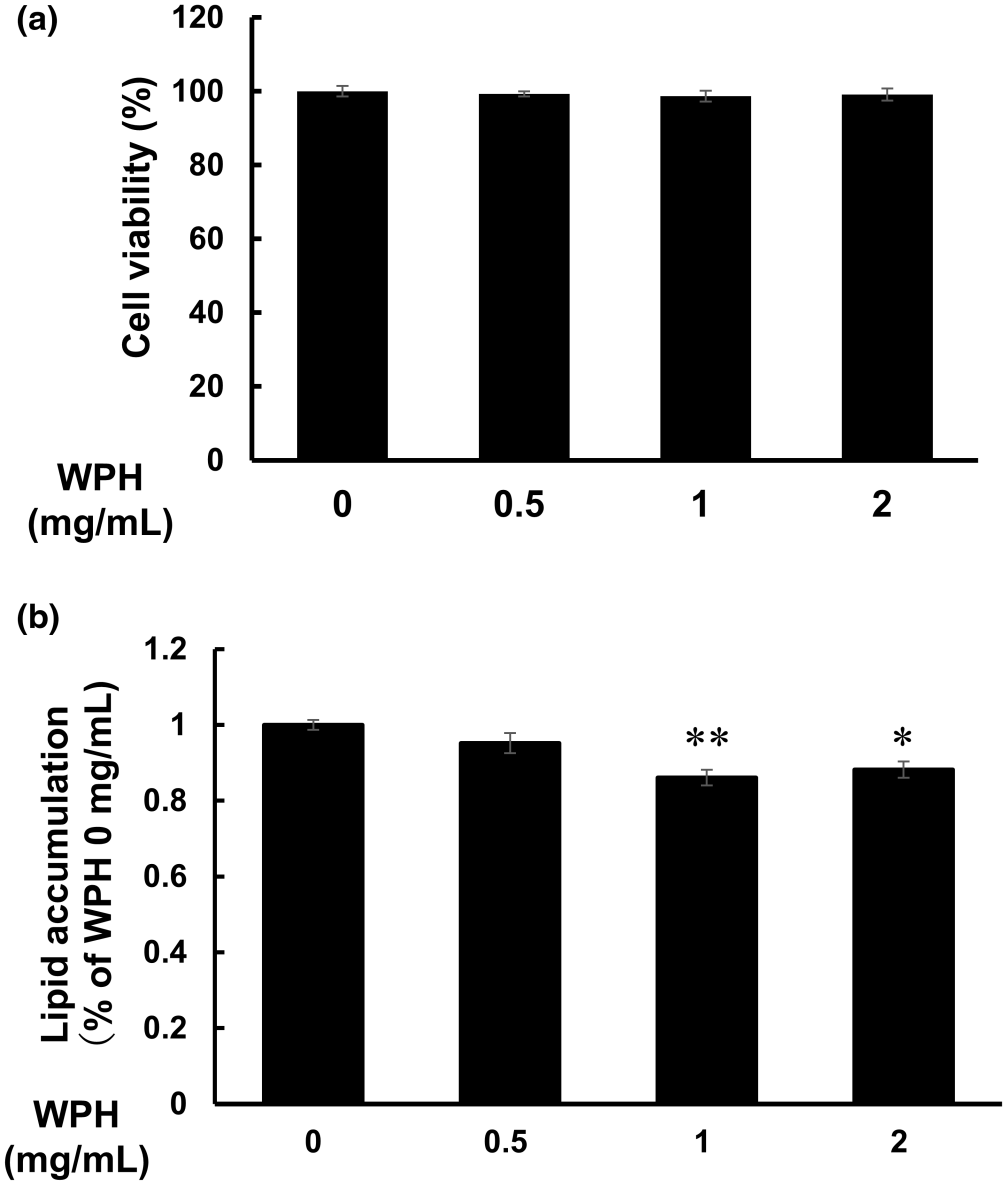
Effects of WPH on lipid accumulation in 3T3‐L1 cells. (a) Oil Red O staining of lipid droplets in 3T3‐L1 cells. (b) Cell viability of 3T3‐L1 cells following treatment with WPH was determined through the WST‐8 assay. Data are presented as mean ± SEM (*n* = 3; *p < 0.05, **p < 0.01).

We initially examined the effect of WPH on cell viability through the WST‐8 assay. 3T3‐L1 preadipocytes were treated with 0.5, 1, and 2 mg/mL WPH in GM for 48 h. The results indicated that WPH treatment did not affect the viability of 3T3‐L1 preadipocytes at the indicated concentrations (Figure 1a).

To investigate the effect of WPH on adipogenesis, 3T3‐L1 cells were differentiated for 6 days with WPH. Intracellular lipid accumulation was assessed using Oil Red O staining. WPH at doses of both 1 and 2 mg/mL reduced the lipid accumulation when compared with the control treatment during adipocyte differentiation (Figure 1b).

### 3.2 Purification of WPH

**FIGURE 2:**
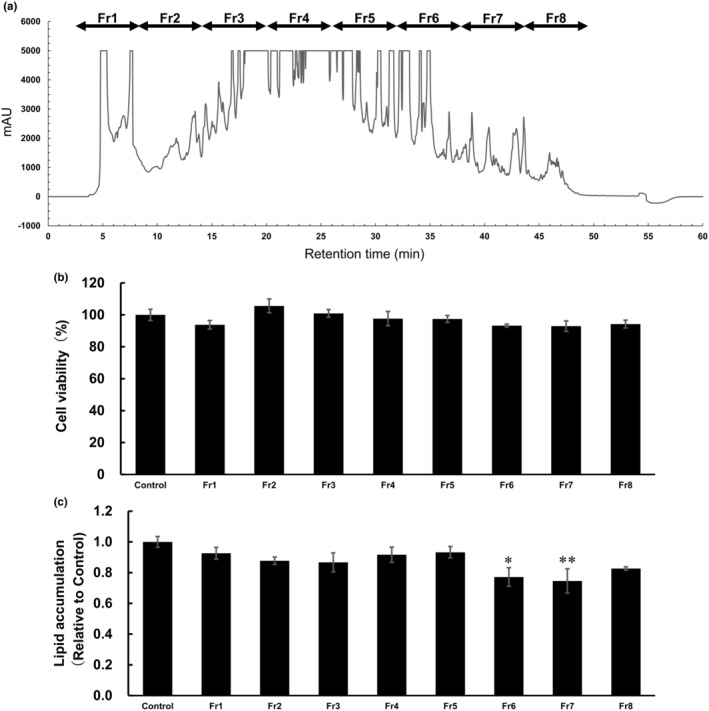
Fractionation of WPH using HPLC and effects of the purified fractions on lipid accumulation and viability in 3T3‐L1 cells. (a) HPLC separation diagram of WPH. (b) Cytotoxicity of Fr1–Fr8, added at a concentration equivalent to 1 mg/mL of WPH, in 3T3‐L1 cells through the WST‐8 assay. (c) Oil Red O staining of lipid droplets in 3T3‐L1 cells treated with Fr1–8 at a concentration equivalent to 1 mg/mL of WPH. Data are presented as mean ± SEM (*n* = 3; *p < 0.05, **p < 0.01).

To identify lipid accumulation‐suppressing peptides, WPH was first fractionated using HPLC equipped with a C18 column, and eight fractions (Fr1–Fr8) were obtained (Figure 2a). The 3T3‐L1 cell viability following treatment with each fraction was assessed using a WST‐8 assay kit. As shown in Figure 2b, Fr1–Fr8, added at a concentration equivalent to 1 mg/mL of WPH, did not affect the viability of 3T3‐L1 cells when compared with the control treatment.

Subsequently, Oil Red O staining was used to assess lipid accumulation in adipocytes. Fr6 and Fr7 had the smallest amount of lipid accumulation among the eight fractions, significantly inhibiting it relative to the control (Figure 2c). These findings suggest that Fr6 and Fr7 influenced lipid droplet accumulation in adipocytes without affecting cell viability.

**FIGURE 3:**
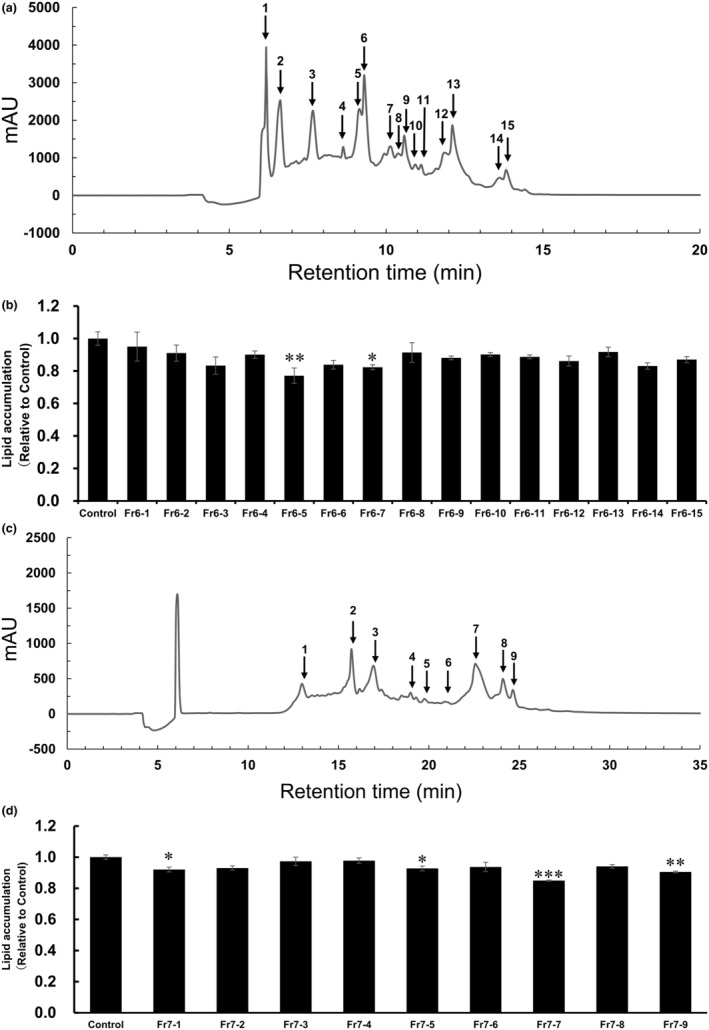
Purification of fractions Fr6 and Fr7 using HPLC chromatography and the effect of purified fractions on lipid accumulation in 3T3‐L1 adipocytes. (a) HPLC using C18 cartridges and individual peaks collected (Fr6‐1 to Fr6‐15). (b) Oil Red O staining of lipid droplets in 3T3‐L1 cells treated with Fr6‐1 to Fr6‐15 at a concentration equivalent to 1 mg/mL of WPH. (c) HPLC using C18 cartridges and the individual peaks collected (Fr7‐1 to Fr7‐9). (d) Oil Red O staining of lipid droplets in 3T3‐L1 cells treated with Fr7‐1 to Fr7‐9 at a concentration equivalent to 1 mg/mL of WPH. Data are presented as mean ± SEM (*n* = 3; *p < 0.05, **p < 0.01, ***p < 0.001).

Fr6 was further separated into 15 distinct peaks (Fr6‐1 to Fr6‐15) using HPLC equipped with a C18 column (Figure 3a). Cells treated with Fr6‐5 or Fr6‐7 at concentrations equivalent to 1 mg/mL of WPH showed lower lipid accumulation than the control (Figure 3b). Fr6‐5 was thus determined as the most active fraction and subjected to further analysis.

Subsequently, Fr7 was separated in the same manner as Fr6. The chromatogram revealed nine major peaks (Fr7‐1 to Fr7‐9) (Figure 3c), and the effects of all nine fractions on lipid accumulation were examined. Among the nine fractions tested, Fr7‐1, Fr7‐5, Fr7‐7, and Fr7‐9 treatment reduced lipid accumulation (Figure 3d). Fr7‐7 had the greatest inhibitory effect among the nine fractions and was therefore selected for further investigation.

### 3.3 Identification and Quantitation of Anti‐Obesity Peptides

Amino acid sequencing and LC–MS analysis were conducted to identify the amino acid sequences of the bioactive peptides which reduce lipid accumulation in WPH, Fr6‐5, and Fr7‐7. These analyses revealed the following: Fr6‐5 contained ALPM and LDQW, while Fr7‐7 contained LKPTPEGDLEIL.

To measure the content of these peptides in WPH, LC–MS analysis was performed. WPH contained 0.2% of ALPM, 0.1% of LDQW, and 0.4% of LKPTPEGDLEIL.

### 3.4 Effect of Anti‐Obesity Peptides on Lipid Accumulation in 3T3‐L1 Adipocytes

**FIGURE 4:**
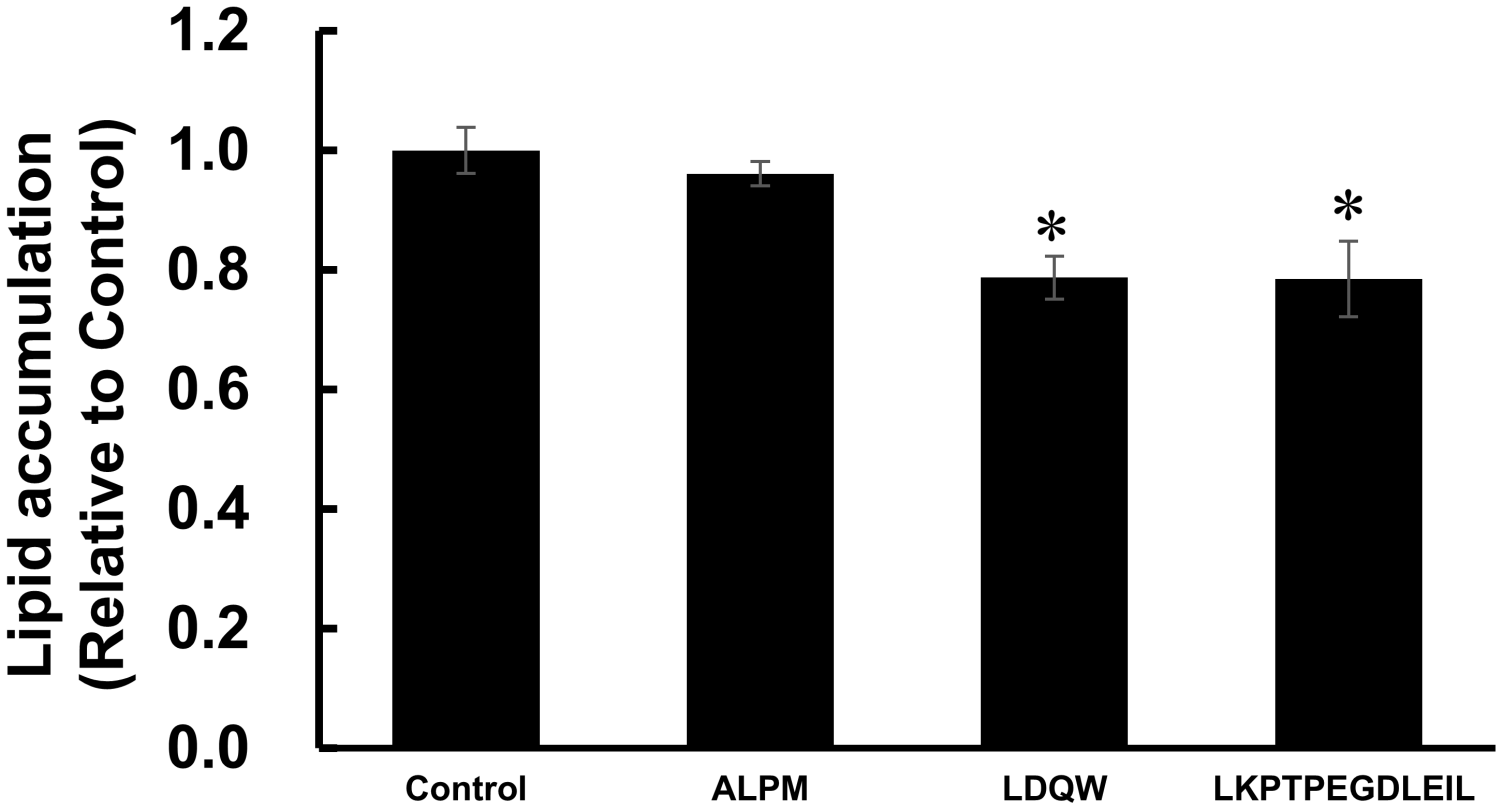
Effect of purified peptides ALPM (2 μg/mL), LDQW (1 μg/mL), and LKPTPEGDLEIL (4 μg/mL) on lipid accumulation using Oil Red O staining. The concentrations of the peptides are equivalent to 1 mg/mL WPH. Data are presented as mean ± SEM (*n* = 3; *p < 0.05).

3T3‐L1 adipocytes were treated with synthetic peptides and subjected to Oil Red O staining to evaluate the effects of ALPM, LDQW, and LKPTPEGDLEIL on lipid accumulation. 3T3‐L1 cells were treated with 2 μg/mL ALPM, 1 μg/mL LDQW, or 4 μg/mL LKPTPEGDLEIL, which are values equivalent to WPH at 1 mg/mL. LDQW and LKPTPEGDLEIL significantly suppressed lipid accumulation in adipocytes compared with the control (Figure 4). Meanwhile, ALPM had no effect on cellular lipid levels, and 1 μg/mL LDQW and 4 μg/mL LKPTPEGDLEIL did not affect cell viability (Figure S1). To examine the dose‐dependent effects of LDQW on lipid accumulation, 3T3‐L1 cells were differentiated at three dose‐dependent concentrations with LDQW. At concentrations of both 1 and 10 μg/mL, LDQW significantly reduced lipid accumulation compared to the control treatment during adipocyte differentiation (Figure S2) without affecting cell viability (Figure S3).

### 3.5 Effect of WPH and Anti‐Obesity Peptides on Gene Expression in 3T3‐L1

**FIGURE 5:**
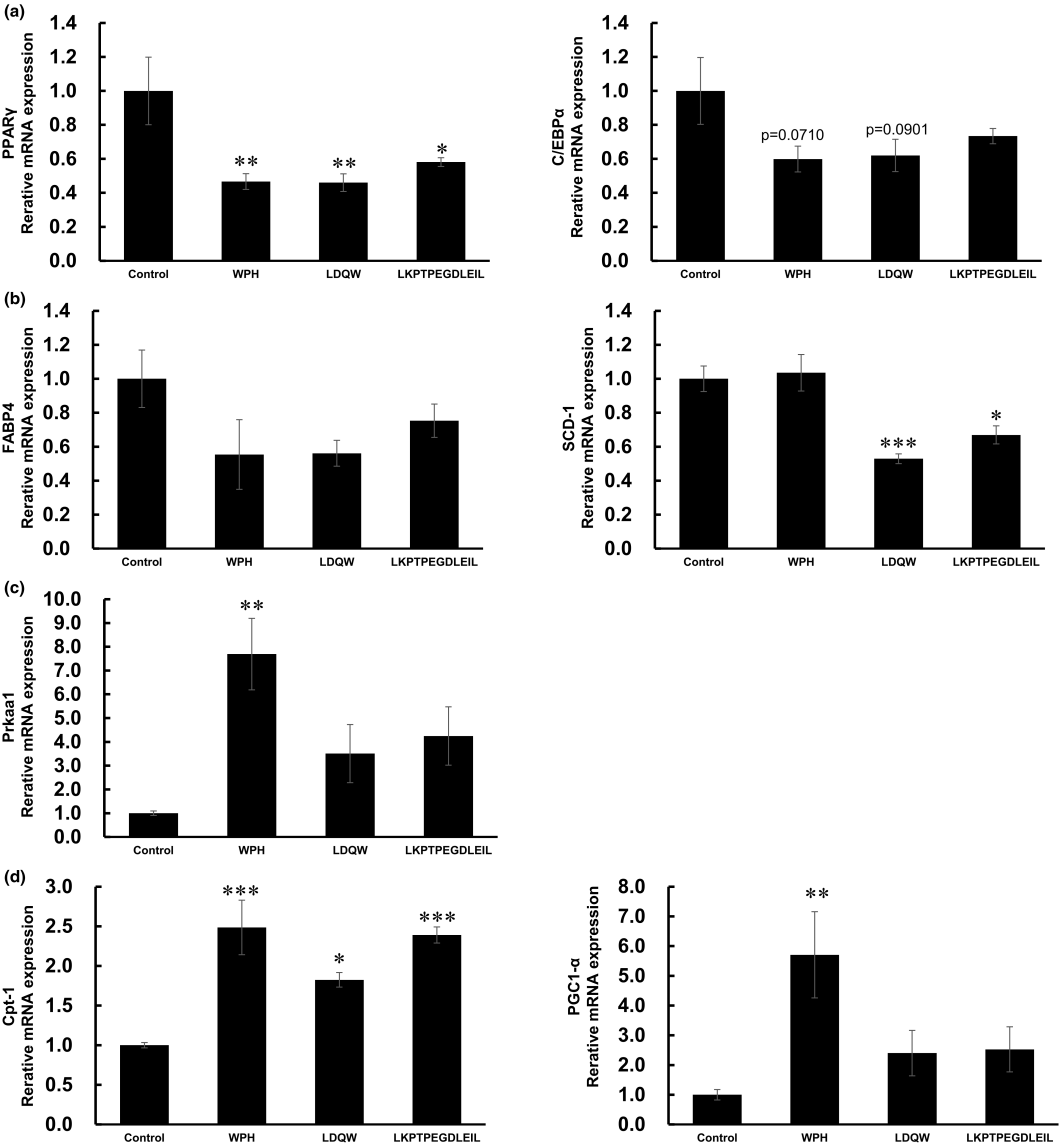
Effects of WPH (1 mg/mL), LDQW (1 μg/mL), and LKPTPEGDLEIL (4 μg/mL) on gene expression. (a) mRNA expression of PPARγ and C/EBPα. (b) mRNA expression of FABP4 and SCD‐1. (c) mRNA expression of PRKAA1. (d) mRNA expression of CPT‐1 and PGC1‐α. Data are presented as mean ± SEM (*n* = 5; *p < 0.05, **p < 0.01, ***p < 0.001).

To investigate whether WPH and WPH‐containing peptides, LDQW and LKPTPEGDLEIL, reduced adipogenesis via the PPARγ pathway, we isolated total RNA from differentiated 3T3‐L1 cells and performed real‐time PCR. WPH, LDQW, and LKPTPEGDLEIL treatments significantly downregulated PPARγ levels, with WPH and LDQW also suppressing C/EBPα mRNA expression (Figure 5a). We then investigated the expression of fatty acid binding protein 4, adipocyte (FABP4), and stearoyl‐coenzyme A desaturase 1 (SCD‐1), which are upregulated by PPARγ and adipogenesis‐related genes. SCD‐1 expression levels were lower in LDQW‐ and LKPTPEGDLEIL‐treated cells than in control cells but not in WPH‐treated cells (Figure 5b). However, there were no significant differences in FABP4 expression (Figure 5b).

We then evaluated whether WPH, LDQW, and LKPTPEGDLEIL could induce the expression of the energy production‐related gene protein kinase AMP‐activated catalytic subunit α 1 (PRKAA1), which activates energy‐producing pathways and inhibits adipocyte differentiation through upstream factors PPARγ and C/EBPs (Lee et al. 2011). WPH treatment significantly increased PRKAA1 levels, whereas LDQW and LKPTPEGDLEIL treatments increased PRKAA1 mRNA expression (Figure 5c). The expression of β‐oxidation‐related genes, including activated AMPK; carnitine palmitoyl transferase 1 (CPT‐1), which is responsible for β‐oxidation and links the transport of long‐chain fatty acids into mitochondria; and PPARγ coactivator‐1α (PGC1‐α), which is a transcription coactivator that controls β‐oxidation increased in the WPH treatment compared with the control. LDQW and LKPTPEGDLEIL treatment increased CPT‐1 mRNA expression (Figure 5d).

## 4 Discussion

In this study, we investigated the effects of WPH on adipocyte differentiation via a 3T3‐L1 adipocyte differentiation assay and identified anti‐obesity peptides derived from whey protein. WPH suppressed lipid droplet accumulation in 3T3‐L1 cells stimulated to differentiate into mature adipocytes. Moreover, we identified two anti‐obesity peptides, LDQW and LKPTPEGDLEIL, using amino acid sequences and LC–MS. These peptides suppressed the differentiation of 3T3‐L1 cells. LDQW exhibits antioxidant (Sadat et al. 2011) and ACE‐inhibitory effects (Xie et al. 2022). LKPTPEGDLEIL has been shown to inhibit DPP‐IV (Lacroix and Li‐Chan 2014). However, to the best of our knowledge, the effects of these peptides on lipid accumulation have not been reported. We identified a new function of LDQW and LKPTPEGDLEIL. Alternatively, to exert their effects in vivo, bioactive peptides have to be absorbed in the intestine. Confirming the anti‐obesity effects of LDQW and LKPTPEGDLEIL would require further in vivo study. Hong et al. reported that peptides shorter than hexapeptides can enter circulation after intestinal absorption (Hong et al. 2016). LDQW and LKPTPEGDLEIL are 4 and 12 amino acids long, respectively. In terms of the absorption kinetics, LDQW may be more effective than LKPTPEGDLEIL in vivo. The absorption kinetics of these peptides in vivo should be determined in the future.

We investigated the mechanism through which WPH, LDQW, and LKPTPEGDLEIL inhibit lipid accumulation by examining the mRNA levels of master adipogenic transcription factors PPARγ and C/EBPα as well as of adipocyte marker genes FABP4 and SCD‐1, which act downstream of PPARγ. Previous studies have revealed that PPARγ and C/EBPα play important roles in adipogenesis and lipogenesis and in regulating the expression of adipocyte markers such as FABP4 and SCD‐1. We found that WPH, LDQW, and LKPTPEGDLEIL suppress the expression of PPARγ, and WPH and LDQW tend to suppress C/EBPα expression. These findings suggest that the suppressive effects of WPH, LDQW, and LKPTPEGDLEIL on lipid accumulation in 3T3‐L1 adipocytes are attributable to the downregulation of adipogenic and lipogenic transcription factors. In previous studies, bioactive peptides derived from tuna fish and hazelnuts suppressed adipogenesis by inhibiting PPARγ and its target genes (Kim et al. 2015; Wang et al. 2020). We found that the treatment of preadipocyte cells with the WPH peptides prevented differentiation by reducing the expression of adipogenic transcription factors PPARγ and C/EBPα.

In the present study, we observed that WPH exhibited a statistically significant effect on lipid accumulation, which was relatively weaker than its effect on gene expression. Factors such as protein expression levels and post‐translational modifications might also influence the phenotype. The limited change in lipid accumulation despite the pronounced effect on gene expression could be attributed to the fact that the changes in these factors were not substantial. As we did not investigate protein expression levels and post‐translational modifications, future studies will be required to confirm how WPH affects these factors.

WPH and β‐lactoglobulin, a component of whey protein, hydrolysate stimulate glucose uptake and glycogen synthesis via AMPK activation (Ichinoseki‐Sekine et al. 2015; Tsuda et al. 2017). Therefore, we also examined the expression levels of genes associated with fatty acid metabolism. WPH upregulated the expression of AMPKα1 (PRKAA1) as well as the expression of AMPK downstream genes, PGC1‐α and CPT‐1, all of which involved in β‐oxidation. However, neither peptide significantly increased AMPK expression. WPH not only inhibited adipocyte differentiation but also promoted fatty acid β‐oxidation. As we did not investigate AMPK phosphorylation, future studies will be required to confirm whether WPH affects AMPK phosphorylation and whether the suppressive effect of WPH on lipid accumulation is mediated through the suppression of PPARγ expression via AMPK phosphorylation.

The results of this study revealed that treatment with WPH, LDQW, and LKPTPEGDLEIL resulted in the downregulation of adipogenesis‐related genes. However, the effects of the identified peptides on some gene expression levels were weaker than those of WPH, and the downstream gene expression patterns were different. Furthermore, only WPH induced significant expression changes in genes related to lipid β‐oxidation. These findings indicated that LDQW and LKPTPEGDLEIL do not regulate lipid metabolism‐related genes on their own but in cooperation with each other and with other peptides in WPH. To further clarify the effect of WPH to utilize it as an effective anti‐obesity functional food, investigations of other peptides and evaluations of the effects of the peptides on lipid metabolism should be performed.

We report two anti‐obesity peptides, LDQW and LKPTPEGDLIEL, derived from WPH. WPH, LDQW, and LKPTPEGDLEIL reduced neutral lipid accumulation in 3T3‐L1 preadipocytes. The inhibitory effect on lipid accumulation is mediated through the suppression of adipogenesis‐related genes, including PPARγ. Although more research is required to validate the beneficial effects of lipid metabolism regulation in vivo or in clinical trials, this study presents preliminary findings for the development of new therapeutic techniques for preventing obesity and other related diseases, such as diabetes, hyperuricemia, and cardiovascular disease.

## Author Contributions

**Yuma Hirose:** conceptualization (equal), data curation (equal), formal analysis (lead), investigation (lead), methodology (lead), visualization (lead), writing – original draft (lead), writing – review and editing (equal). **Masaki Kurimoto:** conceptualization (equal), data curation (lead), methodology (equal), project administration (equal), resources (equal), writing – original draft (equal), writing – review and editing (equal). **Naoki Yuda:** conceptualization (lead), funding acquisition (equal), methodology (equal), project administration (lead), resources (lead), writing – review and editing (lead). **Miyuki Tanaka:** conceptualization (equal), funding acquisition (lead), supervision (lead), writing – review and editing (equal).

## Ethics Statement

The authors have nothing to report.

## Consent

All participants in the study provided informed consent.

## Conflicts of Interest

This work was supported by the Morinaga Milk Industry Co. Ltd., Tokyo, Japan. Y.H., M.K.,N.Y., and M.T. are employed by the Morinaga Milk Industry Co. Ltd., Tokyo, Japan. There are no other conflicts of interest.

## Supporting information


Data S1.

## Data Availability

The authors elects not to share data.
