# Supplementary material for: Identification of Whey Protein‐Derived Anti‐Obesity Peptides Through 3T3‐L1 Adipocyte Differentiation Assay
Source: Food Sci Nutr. 2024 Oct 21;12(11):9641–50. doi: 10.1002/fsn3.4529 (PMC11606838; doi:10.1002/fsn3.4529)

Supplementary Figure 1. Cytotoxicity of LDQW and LKPTPEGDLEIL in 3T3-L1 cells through the WST-8 assay. Data are presented as mean ± SEM (n = 3).


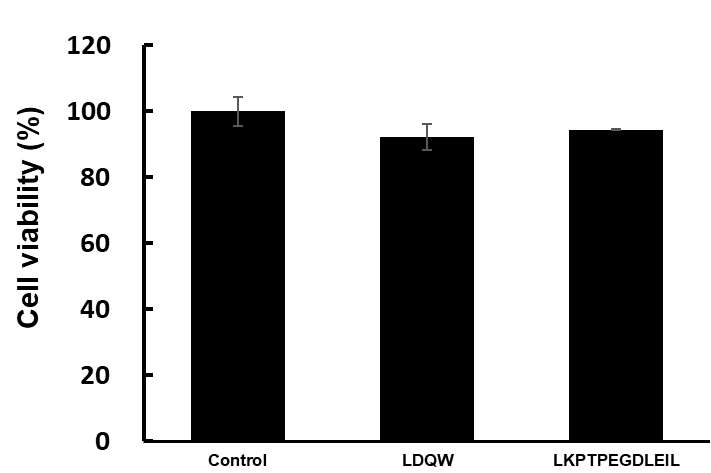


Supplementary Figure 2. Dose-dependent effects of LDQW on lipid accumulation, assessed using Oil Red O staining. Data are presented as mean ± SEM (n = 3; *p < 0.05).


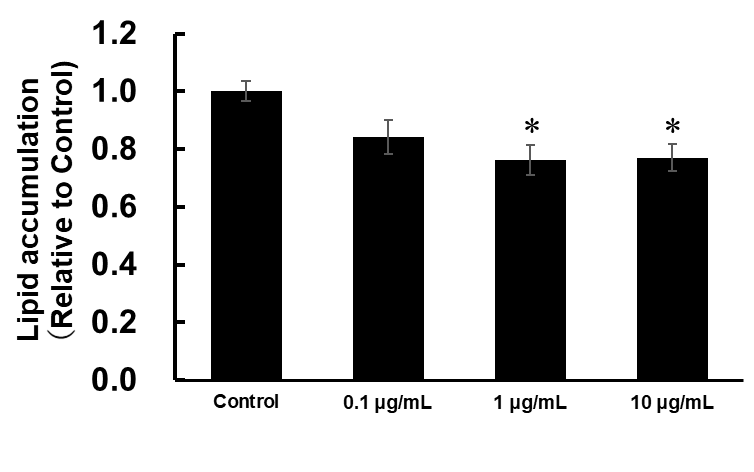


Supplementary Figure 3. Cytotoxicity of LDQW in 3T3-L1 cells through the WST-8 assay. Data are presented as mean ± SEM (n = 3).


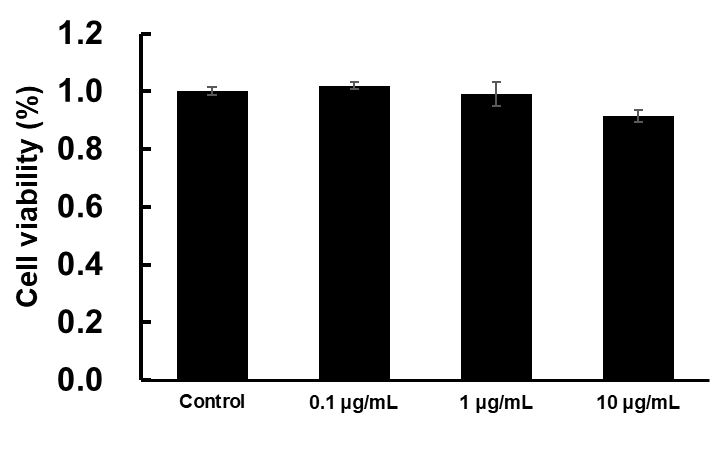

Supplement: Supplementary file 1 — Data S1. [file FSN3-12-9641-s001.docx]
